# Supplementary material for: Patterns of Intron Gain and Loss in Fungi
Source: PLoS Biol. 2004 Nov 30;2(12):e422. doi: 10.1371/journal.pbio.0020422 (PMC532390; doi:10.1371/journal.pbio.0020422)
Supplement: Table S1 — Also available at http://genes.mit.edu/NielsenEtAl/. (4.3 MB ZIP). [file pbio.0020422.st001.zip › NielsenEtAl/html/641.html]

AN1625.1.NCU02321.1.MG00268.1.FG10498.1


```
 CLUSTAL W (1.82) Multiple Sequence Alignments - Introns Inserted


Sequence 1: NCU02321.1	681 aa
Sequence 2: MG00268.1	714 aa
Sequence 3: FG10498.1	651 aa
Sequence 4: AN1625.1	680 aa
Alignment Length: 730 aa
Number Identitical Residues: 153 aa
Alignment Score (without introns) 10250


MG00268.1 	MDAGTNP~SSRAGEMPASADANPAAGDQEQGTGIVDLLPIP-DNIQPVTDPTR--KETSN
NCU02321.1	MLYENLT~EKRTDPLTGEKEENPITGDTGPGTGLQDLLPLPAEDAQDILQSKKAFDKSAG
FG10498.1 	MTRQLPS2RRFSTGVPSEPE---------------------------IVEQHQ-------
AN1625.1  	--MSDAP~DSLSSDKAKEEEINAAFAGKNPKK----------FNPNDPSDAAN-------
          	      .    :   . . : ..  ..    .           . .   :  .       

MG00268.1 	TLEGDVTLSHALASTDKDNHSTNGVLE--LNHGRTEAADLGWHEKKQNIAEPLIGGLSNE
NCU02321.1	GLEDGHTLSHALATED---HKLKGAAQ--LKT-EKEVSNLGWNEPKREIAAPLVGGIDNE
FG10498.1 	--EEAPTVSHALAEESK-NFEEKGAAQ--LEHGDVEVKNLGWNESPCAVPH-LVGGLKND
AN1625.1  	--YNGPTDSHILSQTKPQDNEAKGLAHRAGENEPGQVSDIGWGSSD-TVQERVVPGLSNE
          	      * ** *:  . .. . :*  . : :    :. ::** .    :   :: *:.*:

MG00268.1 	ELWILVRRFNK0QMYHVKA~SPYPVAGNLDLNFAEEEEFSPDKLRAKLERLYTTVIVGLL
NCU02321.1	ELWMLVRRFNK~ASKSLTI1FT-PVPGGLDLNTAEEEEFSPDKMRANIERLYTTVVVGIL
FG10498.1 	DVWTLIRRFDK~QVFYVKS~IQEPPLGMLDMNIADDEEFSPEKLRAQIERLYVSVLVQLF
AN1625.1  	DLFMLIRRFNK0QIYNVKA~VPDAPLQGLDLVRAEDDHFSPDKLRATLERFYTTIIIGVT
          	::: *:***:*     :.     .    **:  *:::.***:*:** :**:*.:::: : 

MG00268.1 	AT~VKHVARLRSWRESRRTACFCA0AYVFAWTFDCLVFLLSATVLTLIVYAPSRAFLFPP
NCU02321.1	AA~VKHIARLRSWREKERTAWFAG~VYFVAWLFDFIMPLITGVIIALIVHPPTRELLFPP
FG10498.1 	AF~YKQIIRLRSWKETQRTSAFLG~VYTLAWLLDILGPTLIIFLMILIWSPDARNICFPG
AN1625.1  	AF0HEG-----------RADQDVQ~VYFTAWLRDLLIPTIFTTLLTLVLYPPSRKFLFPP
          	*   :            *:      .*  **  * :   :   :: *:  . :* : ** 

MG00268.1 	APMSLVSSKHGGVQKPKSGVLGSHDSVTGAPENHKGEAVEQEASNFVNGMTS~VALSSAV
NCU02321.1	APIALVDSKTGGVQKPWAGTVGSKDSATGAPENHKGEAVEQEADNLVHGLAE~LAVSSAA
FG10498.1 	APPSLIDPKTGGVKTPAAGVLASDDSVTGAPEKHKGEAVEQEAHSFVTSLST0IVVSTAA
AN1625.1  	APIALVDTSTGGVQKPKAGVLGSHDSMTGAPEKYKGEAAEQEASNLMAGIAT~VAVGSAT
          	** :*:... ***:.* :*.:.*.** *****::****.**** .:: .::  :.:.:*.

MG00268.1 	GKHPQDDA~DGEDGALDSAGGPDPTTIAVGTADAREKAQGKDTDASKDKTKVPMETAMWT
NCU02321.1	GKHPAGDV1Q-------------------------------------DKTKEPMELAMWA
FG10498.1 	GKHPQTDP~D------DDSAAPDPTKLTEEATEARQKSVGENPHAEHNRAKKPVSNAVWD
AN1625.1  	GKHDQGNP~EG---APMESKVPDATDIVAKSADAQAAANGEVPAETHDKTREPMKQTVLN
          	***   :  :.   :  .:  ...:     ::.:   : .. .    :::: *:. ::  

MG00268.1 	KMRPIMHAITEVTDTWERFAN2ALSPTRPFPQEVARLRLGTLVLPVVAASLLVSSYVFVK
NCU02321.1	KLRPVMQVIGNISDNWERLAN2AISPTPPFPKDTHRLRLAILVIPLFGASLLVTSYMFVK
FG10498.1 	KARPTMHMMADFVDTWERFGN~ALSPAAPFHCHRPKLILIGVLVPMLLGSYFTSPYMAMK
AN1625.1  	SADQAMRVMADVIDTWERFAN2ALSPTPPFSMMTPRLRLAGVLASGLLVALLTSSYVFMK
          	.    *: : :. *.***:.* *:**: **     :* *  :: . .  : :.:.*: :*

MG00268.1 	AITAGIGFVFFGEPIITRGLNWLNRTVPNWQKLLELRN2TALKGVPTNAQLTITLLRIGE
NCU02321.1	SLTFFLGFGMFGDPVVRPGMEYLNRVVPDWQKLLELRN~TLLKGVPTNAQLTLTLLRIGE
FG10498.1 	GMGFGAGFGFFGDPIITPALNFVNRTYPRWEKYVELRN~TILRGVPTNAQLAITLLRIGE
AN1625.1  	IATFSVGLAFFGDPLIQRGIQYLNREYPHWKELSQLQN2TLLKGVPTNAQLTLTLLRIGE
          	      *: :**:*::  .::::**  * *::  :*:* * *:********::*******

MG00268.1 	NNKAPVPPPPRITDGPPDQPAH-----VTDEHLRATGAEPPLN~ATAEELDEAMAHNPRT
NCU02321.1	ANKAPLPPVPRVIEQPVQDPANGPAPLDSEAQMRAEAAAHPLE~ASPSELDEAAHTDPKD
FG10498.1 	KNKAPLPPPPTSDVPPP-----------TVPHETAGEGLEHLG1VSQAEIAAAVQPDPNI
AN1625.1  	ANAAPLPPPPSGSVEKAP-----------SQPASIDHDTLKLG~AAPNEIDRAAAPEPEH
          	 * **:** *                               *  .:  *:  *   :*. 

MG00268.1 	KYETDGTDIEASKSSKHGKVGSKIMGLFKGTSKGGVKTAIGTDTMRAKVLGSHPAKERLG
NCU02321.1	DLRDAGFDVDSHPKNKQTKKSHKVLGWIKNVTAGVVHTGGATDHVRAK-LGAHHAKDRLG
FG10498.1 	LNEDDENDEEKSK----HKKSHRIMNFIRTTAKGGVTTALAADKAKAK-AGAPHAKNRLG
AN1625.1  	PNMHQQEKAEFEKPKQKKGFGAKLLSFFRGTTAAGIESKLAISHARAE-IGNLHAKNQKG
          	       . :    ..    . :::. :: .: . : :  . .  :*:  *   **:: *

MG00268.1 	VVPPKKGETDIKTGPVEFKARHEGRKGRIYIS--ATPALVGFSTKEGDSDDGHKAS--ED
NCU02321.1	VVPPK--DEIPVSGPVRFDCRYEGKKGHCYISTAATIPSVAFVAKKDESSTTVIAGKERE
FG10498.1 	VVKGE--QPNPITGPIRFPARYKGKKGHAYITATATTPAVSWTIELED------------
AN1625.1  	VLRKK-GLETLPMGPVEFDARYQGHRGMAIIDSSHEPALLYFTTDETAQLGDYRME-SRK
          	*:  : .      **:.* .*::*::*   * ::   . : :  .   .        . .

MG00268.1 	VVHPEWSVAVSEIAELRKFGGYGWKAKLIVGWSLER-KVEDGLEIIT-HSGKSFKVTACP
NCU02321.1	DLKPMWTVAVADIMELKKVGGYGWKAKIVVDWSMER-EVKDGLEIVT-MEGKRFRVTACP
FG10498.1 	-LKPAWTVSIGEIDELRKMGGLGWKSKIIVGWAMGS-EIVDGLIIKT-KDGQEHHLTAVS
AN1625.1  	NGSVYFDIPVSDIKEMRKIGGMGWKGKLVTGWAVGGKEVVDGIVVKGNHSHQEFQLTAVT
          	     : :.:.:* *::*.** ***.*::..*::  .:: **: :  . . : .::** .

MG00268.1 	LRDELFNRLVAMGGQKWVAW
NCU02321.1	LRDELFNRLVAMGGQRWEAW
FG10498.1 	MRDQLFNRLISMGSQMWEAW
AN1625.1  	KRDELFNRLVAIDGQVWNSF
          	 **:*****:::..* * ::
```
